# Significant Bradycardia in Critically Ill Patients Receiving Dexmedetomidine and Fentanyl

**DOI:** 10.1155/2017/4504207

**Published:** 2017-09-06

**Authors:** Channing Hui, Maria Cardinale, Balaji Yegneswaran

**Affiliations:** ^1^Rutgers-Robert Wood Johnson Medical School, Department of Emergency Medicine, One Robert Wood Johnson Place, MEB 389, New Brunswick, NJ 08901, USA; ^2^Ernest Mario School of Pharmacy, Rutgers University, 160 Frelinghuysen Rd., Piscataway Township, NJ 08854, USA; ^3^Saint Peter's University Hospital, 254 Easton Ave., New Brunswick, NJ 08901, USA

## Abstract

**Purpose:**

To report a case series of three patients who developed significant bradycardia while receiving the combination of dexmedetomidine and fentanyl for sedation and analgesia.

**Materials and Methods:**

This is a case series of patients obtained from a mixed medical, surgical, and cardiac ICU in a community teaching hospital. Three intubated patients receiving fentanyl and dexmedetomidine infusion developed sudden bradycardia requiring intervention. In all three cases, adjustments to therapy were required.

**Results:**

All three patients experienced significant bradycardia, with a heart rate less than 50 bpm, and one patient briefly developed asystole. In Case  1, the fentanyl infusion rate was reduced by 67% and the dexmedetomidine infusion rate was reduced by 25%. In Case  2, the sedation was changed to midazolam, and in Case  3, both fentanyl and dexmedetomidine were discontinued. In all three cases, there were no further incidences of significant bradycardia following intervention.

**Conclusions:**

Fentanyl used in combination with dexmedetomidine can result in clinically significant bradycardia. Further study is warranted to identify risk factors and elucidate the mechanisms that result in life-threatening bradycardia.

## 1. Introduction

The current pain, agitation, and delirium guidelines encourage the use of fentanyl with sedation strategies using nonbenzodiazepine sedatives [1]. Dexmedetomidine is an effective sedative that is often used in conjunction with opioids such as fentanyl in the critical care and anesthesia setting. Bradycardia and hypotension are well-known associations with dexmedetomidine, yet no human studies have examined the occurrence of drug-induced bradycardia secondary to the combination of dexmedetomidine and fentanyl. One study demonstrated attenuation of the tachycardia response to flexible bronchoscopy with the combination of dexmedetomidine-fentanyl compared to propofol-fentanyl [2]. Here we report three cases in which a synergistic interaction between fentanyl and dexmedetomidine may have contributed to clinically relevant bradycardia.

## 2. Case Series

### 2.1. Case  1

Patient was a 59-year-old male with a past medical history of cirrhosis, ulcerative colitis, esophageal varices with previous banding, hypertension, and hyperlipidemia. He had advanced alcoholic cirrhosis, with a Model for End-Stage Liver Disease (MELD) score of 17, and was admitted with a large right pleural effusion secondary to ascites. His initial pharmacologic management included furosemide, spironolactone, albumin, midodrine, pantoprazole, lactulose, rifaximin, and antibiotics for a possible pneumonia. During his admission, he received nadolol for portal hypertension, with his heart rate averaging between 55 and 85 bpm, but this was discontinued on hospital day four.

Despite multiple thoracenteses, he ultimately required intubation on hospital day six secondary to worsening respiratory failure. His weight and select values at this time are provided in Table 1. He was initially sedated with propofol alone, which was changed to dexmedetomidine after a few hours due to persistent agitation. Dexmedetomidine was titrated to a Richmond Agitation Sedation Scale (RASS) score of −2. During this time, his heart rate remained between 50 and 65 bpm. Approximately 30 hours later, while receiving dexmedetomidine 0.4 mcg/kg/hour, fentanyl was added at 25 mcg/hour for an elevated pain assessment score. As fentanyl was titrated up to 150 mcg/hour, his heart rate decreased into the range of 40 to 47 bpm. Once the fentanyl rate was decreased to 75 mcg/hour, the heart rate began to increase to 50 bpm. As both infusion rates were decreased, the heart rate steadily improved further. Ultimately, the patient was maintained on dexmedetomidine 0.3–0.6 mcg/kg/hour and fentanyl 50–100 mcg/hour, and his heart rate remained between 55 and 70 bpm. His blood pressure remained stable throughout the entire duration of mechanical ventilation. The patient remained intubated until hospital day 11, when he again became agitated, requiring dexmedetomidine to be increased from 0.5 mcg/kg/hour to 0.6 mcg/kg/hour and fentanyl to be increased to 100 mcg/hour to 200 mcg/hour. Within two hours, his heart rate decreased from 60 bpm to 48 bpm. His heart rate remained between 45 and 48 bpm until he was transferred later that afternoon to a liver transplant center. The patient had no other medications or precipitating factors that would be expected to contribute to bradycardia.

### 2.2. Case  2

Patient was a 75-year-old male with a history of metastatic breast cancer, cholangiocarcinoma, atrial fibrillation, and hyperthyroidism who was initially admitted to the hospital with abdominal pain and found to have a yeast infection in his peritoneal fluid. After seven days in the hospital, he was transferred to the ICU for septic shock due to* E. coli *bacteremia. Despite vasopressor therapy, the patient remained in a persistent shock state and subsequently developed multiorgan failure. Upon ICU admission, the patient was intubated for respiratory failure and initially received propofol and fentanyl for sedation and analgesia but was switched to dexmedetomidine alone due to hypotension. Throughout his ICU course, he required continuous renal replacement therapy and total parenteral nutrition. An echocardiogram from two months prior demonstrated an ejection fraction of 50% and normal diastolic function. Dexmedetomidine was titrated up to 0.7 mcg/kg/hour to achieve a goal RASS of −2 with no hemodynamic complications.

After two days of dexmedetomidine therapy, an intravenous bolus dose of fentanyl 50 mcg was given for acute pain, followed by an intravenous bolus dose of metoclopramide 5 mg given 30 minutes later. Within one hour, the patient's heart rate rapidly decreased, leading to a six-second asystolic pause. The pulse spontaneously returned, but the heart rate remained between 30 and 35 bpm for 15 minutes. Dexmedetomidine infusion was immediately discontinued, and the heart rate ultimately improved to normal without intervention. The patient was switched to midazolam infusion, and there were no further episodes. During the time of the asystole, the patient was also receiving rifaximin for a resolving hyperammonemia, famotidine, heparin, cefazolin, insulin, methimazole, metoclopramide, and norepinephrine. Select labs at this time are provided in Table 1. The patient ultimately expired on hospital day 21 due to cardiac arrest following a prolonged course.

### 2.3. Case  3

A 73-year-old male admitted with myasthenia gravis, benign prostate hypertrophy, and hyperlipidemia was admitted with myasthenia gravis crisis. He was initially treated with intravenous immunoglobulin but required intubation on day 2 for airway management and was later treated with plasmapheresis. During his intubation, he was initially sedated with continuous infusions of fentanyl and propofol. On day 4 of his hospital course, he was switched from propofol and fentanyl to dexmedetomidine monotherapy at doses between 0.1 and 0.4 mcg/kg/hr, and his heart rate ranged from 55 to 82 during this time. Due to agitation, he was then switched back to continuous infusions of fentanyl, and propofol was used for sedation and analgesia to maintain a RASS of −2. During this period, his fentanyl ranged from 50 to 150 mcg/hour, and his propofol ranged from 5 to 45 mcg/kg/min. His heart rate throughout this time ranged mainly between 55 and 110 bpm, with multiple excursions to 140 bpm due to severe agitation, for which he was started on quetiapine.

On day 9, dexmedetomidine was again added to his sedation regimen to facilitate extubation and titrated slowly to 0.4 mcg/kg/hour. On day 11 of his hospitalization, while receiving fentanyl 50 mcg/hour, dexmedetomidine 0.3 mcg/kg/hour, and propofol 20 mcg/kg/minute, his heart rate decreased to the mid-40s and ranged between 44 and 51 bpm. In response to the heart rate, fentanyl was discontinued, followed by dexmedetomidine, and finally propofol, all within two hours of each other. The heart rate began to increase back to normal within 30 minutes of discontinuing all three infusions. No other sedative agent was added after this, and the patient was successfully extubated the next day with no further hemodynamic complications and was discharged to a rehabilitation center after a prolonged hospital course. During the bradycardia episode, the patient was receiving methylprednisolone, quetiapine (started four days earlier for agitation), heparin, famotidine, and ceftriaxone and was on his fifth day of plasmapheresis therapy. He did not receive pyridostigmine until the last day of his hospitalization and had no other precipitating factors for bradycardia.

## 3. Discussion

Dexmedetomidine is an *α*_2_ adrenergic receptor agonist, with studies demonstrating a high ratio of *α*_2_/*α*_1_-activity (1620 : 1) [3]. It is selective for G protein-binding adrenergic receptors, including *α*_2A_-, *α*_2B_-, and *α*_2C_- adrenoceptor subtypes. *α*_2A_-Adrenergic receptor agonism provides sedative and antinociceptive properties, while *α*_2B_-adrenergic receptor has vasoconstrictive properties. *α*_2C_-Adrenoceptor agonism has a variety of effects such as regulating dopaminergic and behavioral responses [4]. The presence of *α*_2_ adrenergic receptors in the presynaptic and postsynaptic terminals contributes to its sedative and analgesic properties. The activation of the presynaptic terminal leads to decreased transmission of pain signals, and activation of the postsynaptic terminal inhibits the sympathetic nervous system, elucidating its hypotensive and bradycardic effects [5]. The activation of *α* adrenergic receptors causes hyperpolarization of membranes from potassium channel activation, which decreases norepinephrine release. This is significant in the locus coeruleus, which provides adrenergic innervation to the brain and associated with brain functions such as arousal and sleep [4].

Data regarding the association between dose of dexmedetomidine and the incidence of bradycardia seem inconclusive. One retrospective study that compared low- and high-dose dexmedetomidine in ICU patients showed no significant difference in the incidence of hypotension and bradycardia [6]. Another study that measured plasma concentrations of dexmedetomidine demonstrated an indirect relationship between concentration and heart rate [7]. Lastly, a retrospective cohort study identified risk factors such as advanced age and low baseline arterial blood pressure to be associated with hemodynamic instability with dexmedetomidine but did not identify doses greater than 0.7 mcg/kg/hour to be an independent risk factor [8]. Interestingly, cardiac or sedative medications given concurrently with dexmedetomidine were also not associated with higher rates of instability [8].

Fentanyl exerts its effects by binding to opioid receptors in the brain and spinal cord [9]. Fentanyl is known for its relatively favorable hemodynamic profile, especially in the critically ill when cardiovascular depression is undesired [10]. Although uncommon, fentanyl-induced bradycardia is a known adverse effect, yet the exact mechanism is unknown. A proposed mechanism involves the indirect increase of parasympathetic activity in the cardiac vagal neurons in the nucleus ambiguus. The stimulation of *μ*-opioid receptors in rats by endomorphin-1 postsynaptically via the G protein pathway inhibits calcium currents, leading to increased vagal stimulation to the heart [11]. In addition, fentanyl specifically has been suggested to have an inhibitory role in GABAergic neurotransmission in cardiac vagal neurons, thus increasing parasympathetic activity with induced bradycardia [12].

This case series highlights three critically ill patients who developed significant bradycardia while receiving the combination of dexmedetomidine and fentanyl for sedation and analgesia. According to the Drug Interaction Probability Scale (DIPS), all three cases can be classified as probable drug interactions [13]. Although these drugs individually exert their effects via different pathways, little is known regarding a synergistic or additive effect on heart rate reduction. In 1994, Salmenperä and colleagues postulated that fentanyl may augment the bradycardic effects of dexmedetomidine in a dog model [14]. A shared pathway of *μ*-opioid and *α*_2_ adrenergic receptors is the stimulation of the guanine nucleotide regulatory protein, leading to the decreased levels of adenylate cyclase. The downstream effects include hyperpolarization of the membranes secondary to the efflux of potassium ions [14, 15]. This may explain the additive or synergistic effects of bradycardia.

To our knowledge, this is the first such case series reporting clinically significant bradycardia among humans that appeared to be worsened when fentanyl and dexmedetomidine were given concomitantly. This finding is contradictory to previous reports that concomitant use of sedatives with dexmedetomidine does not increase the risk of hemodynamic instability [8]. Our findings warrant several considerations for the ICU clinician. First, as dexmedetomidine use in critically ill patients continues to expand, clinicians should be aware that significant bradycardia can develop when dexmedetomidine is used in combination with fentanyl, a commonly used analgesic in the ICU. This effect may be exaggerated in patients with liver dysfunction, as both medications are metabolized primarily via the liver. This effect may also be more pronounced in patients receiving prolonged infusions of dexmedetomidine. Although the Food and Drug Administration suggests that continuous infusions of dexmedetomidine should not exceed 24 hours [16], current practice often requires extension of the infusion duration beyond this recommendation. Indeed, for all three cases reported here, significant bradycardia or asystole occurred after dexmedetomidine was used for greater than 24 hours. Lastly, patients receiving fentanyl and dexmedetomidine may also be at an increased risk if they receive other agents that can cause bradycardia, including propofol and metoclopramide, both of which are frequently used in the ICU. Further research is warranted to identify risk factors and elucidate the mechanisms that result in life-threatening bradycardia.

## Figures and Tables

**Table 1:** Select demographic data and labs at time of bradycardia.

	Case 1	Case 2	Case 3
Weight (kg)	77	86.8	93.4
Body mass index (BMI)	29.3	25.1	29.9
Serum creatinine (mg/dL)	1.10	1.78	0.61
Serum CO_2_ (mEq/L)	28	20	27
Blood urea nitrogen (mg/dL)	28	41	24
Albumin (g/dL)	1.9	2.6	4.1
Protein (g/dL)	5.4	5.3	4.6
ALT (units/L)	7	4	48
AST (units/L)	29	37	46
Alkaline phosphatase (units/L)	60	164	74
INR	2.12	1.14	1.03
aPTT (seconds)	37.3	24.1	30.6

ALT: alanine aminotransferase; AST: aspartate aminotransferase; INR: international normalized ratio; aPTT: activated partial thromboplastin time.
